# The first complete mitogenome of Indian star tortoise (*Geochelone elegans*)

**DOI:** 10.1080/23802359.2018.1507656

**Published:** 2018-10-14

**Authors:** Shubhagata Das, Tofazzal Md. Rakib, Md. Shafiqul Islam, Md. Mustafizur Rahaman, Tridip Das, Mohammad Alamgir Hossain, Subir Sarker, Shane R. Raidal

**Affiliations:** a Department of Pathology and Parasitology, Chittagong Veterinary and Animal Sciences University, Chittagong, Bangladesh;; b School of Animal and Veterinary Science, Charles Sturt University, Wagga Wagga, Australia;; c Department of Veterinary Medicine, Kagoshima University, Kagoshima, Japan;; d Veterinary Hospital, Bangabandhu Sheikh Mujibur Rahman Safari Park, Cox’s Bazar, Bangladesh;; e Department of Physiology, Anatomy and Microbiology, School of Life Sciences, La Trobe University, Australia

**Keywords:** Indian star tortoise, mtDNA, *Geochelone elegans*, mitogenome phylogeny

## Abstract

The complete mitochondrial genome of Indian star tortoise (*Geochelone elegans*) was characterized having 16,446 bp nucleotides encoding 37 genes in circular orientation comprising 13 protein-coding genes, 22 tRNA genes and two rRNA genes. The lengths of 12S and 16S ribosomal RNA were 973 bp and 1600 bp. A non-coding control region (D-Loop) of 966 bp was identified between tRNA^Pro^ and tRNA^Phe^ having seven interrupted tandem repeats. A single A + 1 frameshift insertion in the ND3 gene (ND3-174) was also discovered. The complete mitogenome of *G. elegans* would contribute in deeper understanding of the evolutionary dynamics and conservation effort of vulnerable testudine families.

The Indian star tortoise (*Geochelone elegans*) is one of the common species of star tortoises native to the dry and scrub forest areas of India and Sri Lanka and can be easily distinguished from the closely related G. *platynota* (Burmese star tortoise) by the distinctive radiating sunburst or star patterns in the plastron. This species is quite popular in exotic wildlife trade, which is the main reason for its rapid population decline and therefore listed as vulnerable species in IUCN red list (D'Cruze et al. 2016). Habitat loss and poaching that fuels the illegal wildlife trade (Sekhar et al. 2004; D'Cruze et al. 2015; Vyas 2015) are the major threats for this species survival. At present, several partial mitochondrial genome of *G. elegans* has been published (Palkovacs et al. 2002; Gaur et al. 2006), but complete mitochondrial genome sequence hasn’t been reported yet.

A total of 250 star tortoise were confiscated by the Bangladesh customs authority at Benapole land port on 23 May 2010 from an illegal consignment and later on rehabilitated (entry no. 0920/10) in Bangabandhu Sheikh Mujibur Rahman Safari Park at Dulahazra, Cox’s Bazar (24.1700° N, 90.3966° E). In 2015, unexpectedly three quarter of all tortoises died and a thorough post mortem investigation was conducted by a team of pathologist from Chittagong Veterinary and Animal Sciences University (CVASU). For this study total genomic DNA was extracted from the preserved spleen sample (AC. No. DPP/2015/102) of an individual male tortoise. Next generation sequencing (NGS) of the genomic DNA was performed at Annoroad Gene Technology Co. Ltd., China from paired-end library (150bp insert size) constructed using Nextera^®^ DNA library preparation kit (Illumina, San Diego, CA, USA) and A HiSeq4000 sequencing platform. De Novo assembly was performed on the cleaned data using SPAdes assembler (Bankevich et al. 2012). Annotation was performed with MITOS (Bernt et al. 2013), and the protein coding ORFs were further assessed using the Glimmer V. 3.0 (Delcher et al. 2007).

The complete mitogenome was 16,446 bp in length encoding a total of 37 genes in circular orientation comprising 13 protein-coding genes, 22 tRNA genes and two rRNA genes as typically found in all vertebrates. The lengths of 12S and 16S ribosomal RNA were 973 bp and 1600 bp where a non-coding control region (D-Loop) of 966 bp was also positioned between tRNA^Pro^ and tRNA^Phe^ having seven interrupted tandem repeats. The AT and GC contents of this complete mitogenome was 58.70% and 41.30% respectively. Furthermore, a single A + 1 frameshift insertion in the ND3 gene (ND3-174) was also discovered which is a common attribute in mitochondrial genome of a wide range of amphibians and reptiles (Russell and Beckenbach 2008). NCBI nucleotide blast showed 88% pairwise nucleotide identity with different chelonoidis mitochondrial genome. However, Maximum likelihood (ML) tree comprising all publicly available complete mitochondrial genome of turtles and tortoises have positioned the newly obtained mitogenome of *G. elegans* in a monophyletic clade belonging to different tortoise species having maximum bootstrap support with Aldabra giant tortoise (*Aldabrachelys gigantea*; GenBank reference no. NC_028438). The complete mitogenome of *G. elegans* will add valuable information of the genetic diversity within Testudinidae family and might have implication for the conservation of the species.
